# Roles of aminoacyl-tRNA synthetases in immune regulation and immune diseases

**DOI:** 10.1038/s41419-019-2145-5

**Published:** 2019-11-28

**Authors:** Anzheng Nie, Bao Sun, Zhihui Fu, Dongsheng Yu

**Affiliations:** 1 Department of Chinese Medicine, The First Affiliated Hospital of Zhengzhou University, 450000 Zhengzhou, China; 2 Department of Clinical Pharmacology, Xiangya Hospital, Central South University, 410000 Changsha, China; 3 Hunan Key Laboratory of Pharmacogenetics, Institute of Clinical Pharmacology, Central South University, 410000 Changsha, China

**Keywords:** Immunology, Molecular biology

## Abstract

Aminoacyl-tRNA synthetases (ARSs) play a vital role in protein synthesis by linking amino acids to their cognate transfer RNAs (tRNAs). This typical function has been well recognized over the past few decades. However, accumulating evidence reveals that ARSs are involved in a wide range of physiological and pathological processes apart from translation. Strikingly, certain ARSs are closely related to different types of immune responses. In this review, we address the infection and immune responses induced by pathogen ARSs, as well as the potential anti-infective compounds that target pathogen ARSs. Meanwhile, we describe the functional mechanisms of ARSs in the development of immune cells. In addition, we focus on the roles of ARSs in certain immune diseases, such as autoimmune diseases, infectious diseases, and tumor immunity. Although our knowledge of ARSs in the immunological context is still in its infancy, research in this field may provide new ideas for the treatment of immune-related diseases.

## Facts

- ARSs are involved in a wide range of physiological and pathological processes apart from translation.
- Pathogen ARSs can induce immune responses in the host, which in turn serve as targets for anti-infection.
- ARSs are involved in the maturation, transcription, activation and recruitment of immune cells, thus playing a crucial role in the development of immune cells.
- More importantly, ARSs act as regulators and signaling molecules in various immune diseases, such as autoimmune diseases, infectious diseases, and tumor immunity.

## Open questions

- Can the safety and drug resistance of potential anti-infective compounds that target pathogen ARSs be resolved?
- Does the dysregulation of ARSs in immune diseases affect the synthesis of specific proteins?
- What is the molecular mechanism by which ARSs trigger autoimmune diseases?
- Is there potential for practical clinical applications based on findings concerning certain ARSs in the immunological context?

## Introduction

Aminoacyl-tRNA synthetases (ARSs) are generally considered as “housekeepers” involved in protein synthesis, whose primary function is to catalyze the aminoacylation of transfer RNAs (tRNAs). This enzymatic reaction is conserved and proceeds mainly in two steps^1,2^. First, an amino acid and an adenosine triphosphate (ATP) molecule bind to the active site of the corresponding ARS to form an aminoacyl adenylate (amino acid-AMP) intermediate and simultaneously release a pyrophosphate (PPi). Second, the cognate tRNA binds to the ARS via its anticodon binding domain, and then the amino acid is transferred to the CCA sequence at 3′ end of tRNA with the release of AMP. Finally, the resulting aminoacylated tRNA enters the ribosome during messenger RNA (mRNA) translation. Each ARS molecule and its encoding gene are named in the form of XRS and XARS, respectively, where X represents the single- or three-letter code of the cognate amino acid^3^. To date, 36 ARSs encoded by different genes have been found in human cells, catalyzing the aminoacylation of all tRNAs^4,5^. Among them, 16 ARSs exclusively play a role in the cytoplasm, 17 ARSs exclusively play a role in the mitochondria, and the remaining 3 ARSs are functional in both parts. Notably, glutamyl-prolyl-tRNA synthetase (EPRS) can catalyze the charging for glutamic acid and proline in the cytoplasm, therefore a full set of ARSs responsible for the charging of the 20 canonical amino acids exists in both the cytoplasm and mitochondria.

Actually, many studies have shown that ARSs participate in a variety of physiological and pathological processes through certain non-normative functions such as angiogenesis, post-translational modifications, translation initiation, and autophagy regulation^6–9^. For example, seryl-tRNA synthetase (SerRS) could bind to transcription factor Yin Yang 1 (YY1) to form a SerRS/YY1 complex, which interacted with distal *cis*-regulatory elements and then negatively regulated vascular endothelial cell growth factor A transcription during angiogenesis^10^. Furthermore, ARSs are emerging as multifaceted molecules participating in immune regulation and immune diseases^11–13^. It has been found that the pathogenic variants in lysyl-tRNA synthetase (KARS) cause a progressive leukodystrophy, and the patients exhibit severe phenotypes including developmental delay or arrest, deafness, and immunological abnormalities^14^. Interestingly, diverse autoantibodies against ARSs were found in antisynthetase syndrome (ASSD), suggesting that ARSs are likely to be involved in the development and progression of autoimmune diseases^15–17^. Strikingly, full-length tryptophanyl-tRNA synthetase (WRS) induced immune cells to release inflammatory cytokines and type I interferons (IFNs), thereby playing a role in antiviral immunity^18^. Therefore, studying the biological functions of ARSs in an immune setting will be a promising field. Here, we not only summarize the immune responses induced by pathogen ARSs, but also elaborate the potential anti-infective compounds that target pathogen ARSs. Meanwhile, we focus on the functional mechanisms of ARSs in immune cell development, as well as in multiple immune diseases, such as autoimmune diseases, infectious diseases, and tumor immunity.

## Pathogen ARSs in immune responses and anti-infective therapies

### Pathogen ARSs induce immune responses

The innate immune response helps the body resist deviant native cells and foreign organisms, which is the key to maintaining health. Typically, each person may be infected with hundreds of pathogens during their lifetime, which will not cause diseases under normal immune function conditions. New evidence suggests that pathogen ARSs are associated with host immune responses.

The *Leishmania donovani* genome encoded a single copy of tyrosyl-tRNA synthetase (TyrRS), which was present as an asymmetric pseudo-dimer in vivo^19^. The heterozygous knockout mutants of *Leishmania* TyrRS (*Ld*TyrRS) showed slow growth and reduced infectivity, suggesting that this gene is essential for the development of this parasite. Notably, *Ld*TyrRS molecule could be released from the parasite cytoplasm to the outside of the cell and specifically bind to the host macrophages by its ELR (Glu–Leu–Arg) peptide motif, thereby promoting the secretion of tumor necrosis factor-alpha (TNF-ɑ) and interleukin-6 (IL-6). Malaria parasite TyrRS (*Pf*TyrRS) entered the infected red blood cell (iRBC) cytoplasm and was released into the extracellular medium upon iRBC lysis^20^. Analogously, *Pf*TyrRS bound to host macrophages via its ELR peptide motif and then was internalized, resulting in increased secretion of the inflammatory cytokines TNF-ɑ and IL-6, as well as overexpression of endothelial receptors intercellular adhesion molecule-1 and vascular cell adhesion molecule-1. Furthermore, *Brugia malayi* asparaginyl-tRNA synthetase (*Bm*AsnRS) has also been shown to be closely related to the host immune and inflammatory responses^21–23^. Kron et al.^24^ demonstrated that *Bm*AsnRS activated IL-8 receptors by extracellular domains that differed from those used by IL-8, providing a basis for elucidating the molecular mechanism by which parasite ARSs participated in immune regulation.

In addition to being associated with parasite-induced immune responses, ARSs also contribute to bacterial metabolism and virulence^25–27^. Previous studies have shown that the two-domain lysyl-transferase (mprF)-lysyl-tRNA synthetase (lysU) protein encoded by the lysX gene is used to produce lysinylated phosphatidylglycerol (L-PG) in *Mycobacterium tuberculosis* (*Mtb*), which is one of the basic phospholipids (PLs) of the *Mtb* membrane^28^. The *Mtb* lysX mutant exhibited altered membrane potential, as well as increased sensitivity to cationic antibiotics and peptides. This may be due to a change in the ratio of PLs caused by the lack of L-PG in the mutant, which in turn led to hyperpolarization of the membrane. More importantly, the lysX mutant not only increased the production of pro-inflammatory cytokines in infected macrophages, but also showed growth defects in the lungs of mice and guinea pigs, indicating that lysX function was indispensable for complete virulence.

### Pathogen ARSs serve as anti-infective targets

The structural differences between pathogen ARSs and human cytoplasmic and mitochondrial ARSs provide a broad platform for the development of anti-infective drugs^29–31^. For example, compared with human cytosolic methionyl-tRNA synthetase (MetRS), there were significant differences in the active site and the location of the connective peptide subdomain 1 (CP1) of *Mtb* MetRS^32^. In addition, two amino acid residues were different between the eubacterial/archaeal isoleucyl-tRNA synthetases (IleRSs) and the eukaryotic IleRSs, resulting in the fact that the antibiotic mupirocin only inhibited the IleRSs of eubacteria and archaea, but not the eukaryotic IleRSs^33^. So far, several successful compounds have been discovered for the treatment of infectious diseases such as malaria, cryptosporidiosis, toxoplasmosis, human African trypanosomiasis, and tuberculosis^34–38^.

The natural product borrelidin was a threonyl-tRNA synthetase (ThrRS) inhibitor with various biological functions such as antifungal, antibacterial, antimalarial, and antiangiogenic activities^39^. By analyzing the crystal structures of both human and bacterial ThrRS-borrelidin complexes, the researchers found that a single borrelidin not only occupied three substrate-binding sites for threonine, ATP and tRNA in the ThrRS catalytic domain, but also extended into a fourth orthogonal pocket. At the same time, based on this, we speculate that the multiple biological functions and cytotoxicity of borrelidin are due to its similar interactions with the different species ThrRSs. Herman et al.^40^ discovered that halofuginol selectively inhibited the cytoplasmic prolyl-tRNA synthetase of *Plasmodium falciparum* (*Pf*cPRS). In *Plasmodium berghei*-infected mice, halofoginol was active against both liver and asexual blood stages of the malaria parasite, indicating that this novel compound had high developmental value. Particularly, the incorporation of 5-fluoroimidazo[4,5-*b*]pyridine into the inhibitors of MetRS of *Trypanosoma brucei* (*Tb*MetRS) could partially or completely cure early-stage and modified late-stage *T. brucei* infection mice^41^. At the same time, compounds with this functional group improved the central nervous system bioavailability, suggesting that further fluorination of *Tb*MetRS inhibitors provided a new idea for the treatment of human African trypanosomiasis. In addition, studies have shown that KRS and phenylalanyl-tRNA synthetase (PheRS) can also be used as effective antiparasitic targets^42–44^. Among them, the compound 5 obtained after optimization of a hit molecule could selectively inhibit apicomplexan KRSs^42^. Molecular dynamics simulations showed that this compound had a higher selectivity for parasite KRSs compared to human KRS due to the combination of a more favorable configuration of the binding site and a higher degree of stabilization upon ligand binding in the parasite KRSs.

Besides their antiparasitic effects, ARS inhibitors also played an important role in the antibacterial and antifungal processes^45–48^. By evaluating the inhibitory effect of a series of 3-aminomethyl 4-halogen benzoxaboroles on *Mtb* leucyl-tRNA synthetase (LeuRS), Li et al.^49^ found that one of the compounds, GSK656, was highly selective for *Mtb* LeuRS and had good antitubercular activity and tolerability in the mid-nanomolar range. Moreover, thiazolin-4-one derivatives as WRS inhibitors showed higher activity against Gram positive bacterial strains than Gram negative bacterial strains^50^. Specifically, compounds 3h and 9b had the best antibacterial activity against *Staphylococcus aureus*, while compounds 8, 10, and 9b had the best antibacterial activity against *Escherichia coli*. CRS3123 inhibited protein synthesis in *Clostridium difficile* by targeting MetRS, thereby preventing the bacterial toxin production and sporulation^51^. Noteworthily, in the phase 1 clinical trial of CRS3123, the healthy subjects did not experience serious adverse events and were well tolerated at all doses tested.

## ARSs in immune cell development

**Fig. 1:**
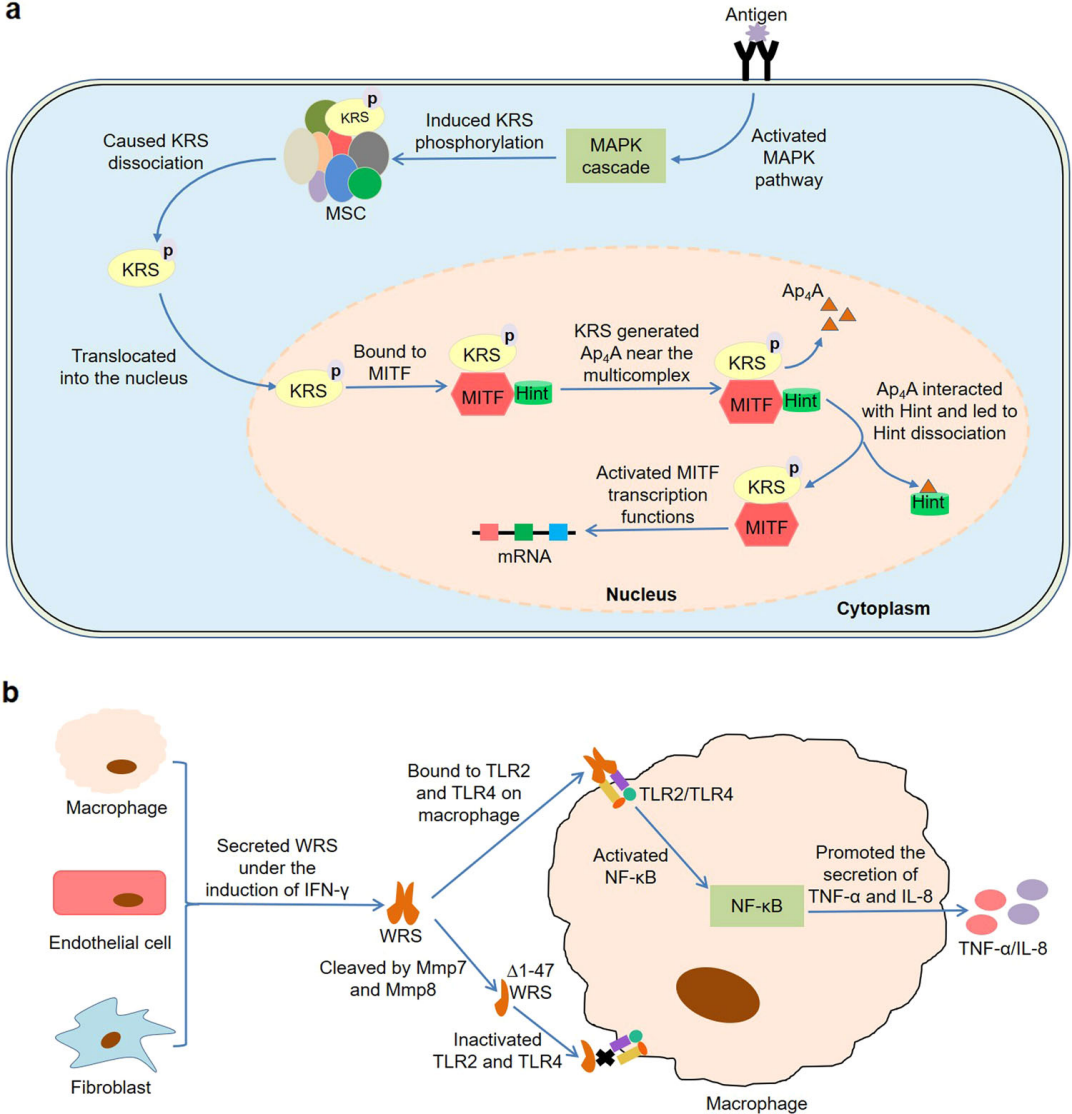
Roles of ARSs in the development of immune cells. **a** KRS is phosphorylated on the serine 207 residue by the MAPK pathway after mast cell activation and then is released from the MSC. The dissociated KRS translocates into the nucleus, binds to MITF, and produces higher levels of Ap_4_A. Subsequently, Ap_4_A interacts with Hint, which in turn results in the dissociation of MITF and the activation of MITF-dependent gene expression. **b** IFN-γ induces the secretion of WRS by human macrophages, endothelial cells, and fibroblasts. Then, the secreted WRS activates NF-κB by binding to TLR2 and TLR4 on macrophages, resulting in secretion of TNF-α and IL-8. Moreover, the N-terminal domain of WRS is cleaved under the catalysis of Mmp7 and Mmp8 to generate ∆1–47 WRS, which does not activate TLR2 and TLR4.

It has been reported that certain ARSs are involved in the development of immune cells (Fig. 1). Previous studies found that many genes were differentially expressed in immature dendritic cells (DCs) derived from peripheral blood monocytes compared with mature DCs induced by lipopolysaccharide^52^. During the maturation process, bruton tyrosine kinase (BTK) region clone 2f10-rpi, proteasome subunit alpha type 3 (PSMA3), transcription factor EC (TFEC) isoform, WRS, and CD63 antigen were upregulated, while neuronal apoptosis inhibitory protein and transforming growth factor-beta (TGF-β)-induced 68 kDa protein were downregulated. Similarly, WRS was specifically expressed during the maturation of blood monocytes to different types of macrophages^53^. Furthermore, Lee et al.^54^ found that microphthalmia transcription factor (MITF) bound to KRS and Hint to form a multicomplex in mast cells, where Hint inhibited the transcriptional activity of MITF (Fig. 1a). When mast cells were activated by IgE-Ag, an endogenous molecule diadenosine tetraphosphate (Ap_4_A) was synthesized by KRS and accumulated intracellularly in the vicinity of the multicomplex. Subsequently, Ap_4_A interacted with Hint, which in turn resulted in the dissociation of MITF and the activation of MITF-dependent gene expression. Further studies showed that KRS in the cytoplasm was phosphorylated on the serine 207 residue by the mitogen-activated protein kinase (MAPK) pathway after mast cell activation and then was released from the multisynthetase complex (MSC)^55^. The dissociated phosphorylated KRS could translocate into the nucleus, bind to MITF, and produce higher levels of Ap_4_A near these molecules. Mechanistically, Ofir-Birin et al.^56^ believed that the conversion of KRS between translation and transcription depended on the transformation of its structure. In quiescent mast cells, KRS stayed in the MSC by binding to the N-terminus of the scaffold protein ARS complex interacting multifunctional protein 2 (p38/AIMP2), thereby maintaining its translational function. After phosphorylation on the serine 207 residue, structural changes disrupted the binding grooves for p38/AIMP2, thereby provoking KRS release and nuclear translocation. Meanwhile, the structural alteration also exposed its C-terminal domain, which in turn triggered the binding to MITF and subsequent generation of Ap_4_A.

During cell competition, dying loser cells secreted TyrRS as a signaling molecule through a c-Jun-N-terminal kinase (JNK) and *Kish*-dependent mechanism^57^. Subsequently, the secreted TyrRS was cleaved into MiniTyr and EMAP fragments by matrix metalloproteinases (Mmps). The latter activated phosphatidylinositol 3-kinase in *Drosophila* macrophages and acted as a chemoattractant to recruit macrophages to loser cells, thereby eliminating these apoptotic cells from healthy tissues. Noh et al.^58^ demonstrated that resveratrol upregulated WRS expression in IFN-γ-induced bone marrow-derived dendritic cells by activating glycogen synthase kinase-3β, thereby regulating CD8^+^ T cell polarization. Under the induction of TNF-α, certain human cells were capable of secreting KRS at levels less than 1% of total cell content^59^. Interestingly, the secreted KRS could specifically bind to receptors on macrophages and peripheral blood mononuclear cells (PBMCs), thus promoting the secretion of TNF-α from these immune cells. Therefore, these two endogenous molecules seemed to form a positive feedback loop between the corresponding cells. Further studies revealed that KRS-induced signal transduction was associated with extracellular signal-regulated kinase (ERK), p38 MAPK, and inhibitory G protein (Gαi). Moreover, the pro-inflammatory cytokine IFN-γ induced the secretion of WRS from human macrophages, endothelial cells, and fibroblasts^60^ (Fig. 1b). Then, the secreted WRS activated nuclear factor-κB by binding to Toll-like receptor 2 (TLR2) and TLR4 on macrophages, resulting in secretion of TNF-α and IL-8. Importantly, the N-terminal domain of WRS was cleaved under the catalysis of Mmp7 and Mmp8 to generate ∆1–47 WRS. This major cleavage product could not activate TLR2 and TLR4, suggesting that WRS mediated the pro-inflammatory response of IFN-γ, and this process could be inhibited by MMPs. Therefore, ARSs are closely related to immune cell maturation, transcription, recruitment, and activation, indicating that these molecules play a crucial role in the development of immune cells.

## ARSs in immune diseases

As mentioned above, ARSs work as regulators and signaling molecules in immune cell development. It is not difficult to imagine that ARSs also function as pleiotropic molecules that regulate various biological processes in immune diseases such as autoimmune diseases, infectious diseases, and tumor immunity.

### ARSs and autoimmune diseases

It is well known that ARSs are often involved in the development of ASSD as specific autoantigens. This disease is a heterogeneous group of autoimmune disease characterized by interstitial lung disease (ILD), myositis, mechanic’s hands, Raynaud’s phenomenon, and arthritis^61^. Up to now, there are mainly eight anti-ARS autoantibodies including anti-histidyl (anti-Jo-1), anti-alanyl (anti-PL-12), anti-threonyl (anti-PL-7), anti-asparaginyl (anti-KS), anti-glycyl (anti-EJ), anti-phenylalanyl (anti-Zo), anti-tyrosyl (anti-Ha), and anti-isoleucyl (anti-OJ) in ASSD^62,63^. Among them, anti-Jo-1 antibody is the most common. A previous study by Stone et al.^64^ found that the levels of anti-Jo-1 autoantibody were modestly correlated with idiopathic inflammatory myopathy activity. Strikingly, the anti-ARS autoantibody specificity was related to the clinical features, disease severity, and even survival of ASSD patients^65–68^. Hamaguchi et al.^69^ discovered that the anti-ARS autoantibodies were generally mutually exclusive, meaning that two or more antibodies rarely appeared in the same ASSD patient. More importantly, they found that the clinical diagnosis of anti-Jo-1, anti-EJ, and anti-PL-7 was mostly polymyositis or dermatomyositis; the clinical diagnosis of anti-PL-12 was mostly clinically amyopathic dermatomyositis or ILD; and the clinical diagnosis of anti-KS and anti-OJ was mostly ILD. Meanwhile, patients with anti-PL-7, anti-EJ, and anti-Jo-1 autoantibodies would develop myositis later if they only showed ILD at the time of onset. Compared with patients without anti-PL-7, Chinese ASSD patients with anti-PL-7 were more likely to develop rapidly progressive ILD and their survival rate decreased more rapidly at the early stage of long-term follow-up^70^. Furthermore, by analyzing the log-rank test and Cox proportional hazards ratio, researchers demonstrated that non-Jo-1 autoantibody patients had worse survival compared with Jo-1-positive patients^71^.

**Fig. 2:**
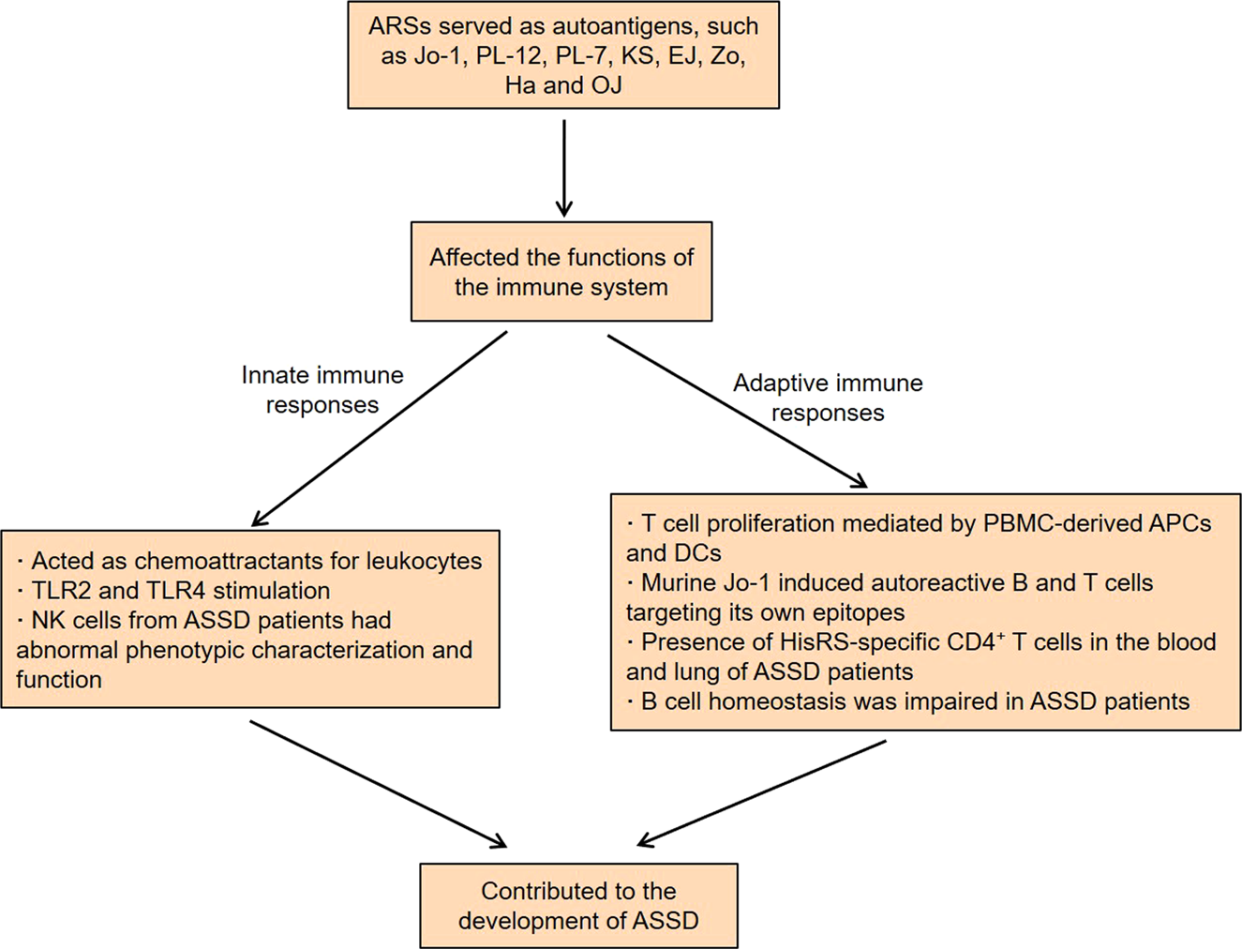
Roles of ARSs in the development of ASSD.

Indeed, successive research has been trying to define the relationship between ARS autoantigens and innate and adaptive immune responses (Fig. 2). Howard et al. revealed that histidyl-tRNA synthetase (HisRS) and AsnRS could act as chemoattractants for leukocytes, while other ARSs without antigenic activity had no similar chemotactic activity^72^. Specifically, these two myositis autoantigens selectively induced migration of lymphocytes, activated monocytes, and immature DCs. Moreover, HisRS induced CC chemokine receptor 5 (CCR5)-transfected cells to migrate, while AsnRS induced CCR3-transfected cells to migrate. Recently, the unique N-terminal extension domain of human AsnRS has been shown to be associated with the CCR3-mediated chemotactic activity^73^. Fernandez et al.^74^ observed that recombinant HisRS provoked myositis in mice via multiple myeloid differentiation primary response gene 88 (MyD88)-dependent TLRs. That is, HisRS-stimulated TLR2 and TLR4 double-knockout mice showed a significant reduction in muscle inflammation, whereas TLR2 and TLR4 single-knockout mice still showed lymphocytic infiltration of muscle tissue. Natural killer (NK) cells from ASSD patients had abnormal phenotypic characterization, such as increased expression of inhibitory receptor Ig-like transcript 2 (ILT2) and differentiation-related receptor CD57, as well as decreased expression of the activating receptor NKp30^75^. Meanwhile, the synthesis of IFN-γ in the IL-12 plus IL-18-stimulating NK cells was significantly impaired, indicating that the cell function was also abnormal. Notably, the infiltrations of NK cells in the lung tissues of ASSD patients were dense and diffuse. These findings suggest that NK cells may contribute to the development and progression of ASSD.

Ascherman et al.^76^ supported that both PBMC-derived antigen-presenting cells (APCs) and DCs mediated peripheral blood T cell proliferation triggered by full-length human Jo-1, but only DCs supported proliferative responses to the Jo-1 fragment^76^. More profoundly, this T cell proliferation was found to be major histocompatibility complex (MHC) class II dependent, indicating the presence of Jo-1-specific T cell responses in Jo-1-positive polymyositis patients. By analyzing the early antibody responses induced by human or mouse Jo-1, researchers found that the B cell and T cell responses to Jo-1 immunization showed significant species-specifity^77^. Of note, murine Jo-1 induced autoreactive B and T cells targeting its own epitopes, and the epitope spreading occurred uniformly 8 weeks after the single immunization, suggesting that autoantibody Jo-1 was able to drive a sustained immune response. When PBMCs and bronchoalveolar lavage fluid (BALF) cells from ASSD patients were stimulated with HisRS or a HisRS-derived peptide (HisRS_11–23_), the expression of CD40L in CD4^+^ T cells from the corresponding compartments was upregulated^78^. Compared with PBMCs, BALF CD4^+^ T cells showed a remarkable Th1 phenotype after stimulation, such as the production of more IFN-γ and IL-2, indicating the presence of HisRS-specific CD4^+^ T cells in the blood and lung of patients with ASSD.

In addition to these possible autoimmune responses, the immune system of ASSD patients also exhibits other abnormalities. Recent studies found that the frequency of CD19^+^CD27^+^ memory B cells in peripheral blood of ASSD patients with anti-Jo-1 was decreased, while the frequency of CD19^+^CD27^−^ naive B cells was increased^79^. Moreover, infiltrating CD20^+^CD27^+^ memory B cells were present in the muscle of anti-Jo-1 patients, indicating that B cell homeostasis was impaired in ASSD. Compared to Jo-1-negative patients, Jo-1-positive patients showed an Fc-glycan profile with less bisected and afucosylated glycans, which was further enhanced in anti-Jo-1 autoimmune IgG^80^. Importantly, the Fc-glycan profile features were correlated with certain clinical and diagnostic information of the patients, suggesting that the specific IgG Fc-glycans might be responsible for the pathogenicity of anti-Jo-1 autoantibodies.

Interestingly, ARSs were also dysregulated in other autoimmune diseases, including multiple sclerosis, rheumatoid arthritis, immune thrombocytopenia, and systemic lupus erythematosus^81–84^. For example, Narasimhan et al.^85^ attempted to predict synovial gene expression by analyzing the characteristics of serum metabolomic profiles of patients with rheumatoid arthritis. They observed that serine/glycine metabolism and aminoacyl-tRNA biosynthesis were related to TNF-α/CD3E and B/plasma cell signatures, indicating that these pathways might be involved in the regulation of lymphocyte functions in the rheumatoid synovium. In general, indoleamine-2,3-dioxygenase (IDO) and WRS are responsible for the metabolism and utilization of tryptophan, respectively, and play important roles in immune regulation^86^. The ratio of serum kynurenine to tryptophan was increased in Graves’ disease patients compared to healthy controls^87^. Further studies found that IDO expression in B cells and DCs was higher than that in healthy controls, but not in CD4^+^ T cells. In contrast, WRS expression in CD4^+^ T cells was higher than that in healthy controls, but not in B cells and DCs. The high levels of WRS in CD4^+^ T cells abolished IDO-mediated immunosuppression from DCs, which might be related to the pathogenesis of Graves’ disease.

### ARSs and infectious diseases

#### ARSs in virus infection

**Fig. 3:**
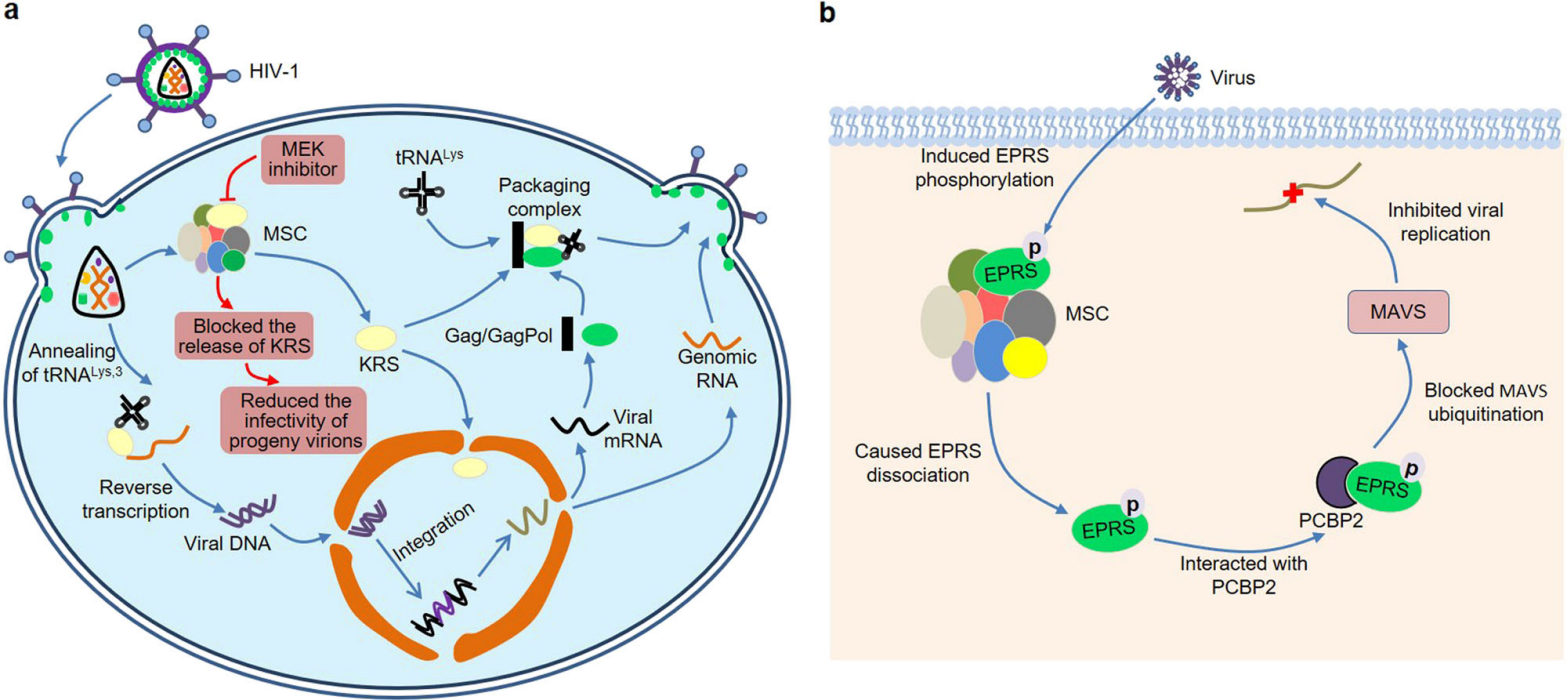
ARSs and virus infection. **a** HIV-1 infection triggers the release of KRS from the MSC to form a free pool of KRS, and then the released KRS is partially transported to the nucleus. Blocking this pathway by the addition of MEK inhibitor reduces the infectivity of progeny virions. Furthermore, KRS binds to a tRNA-like element located near the primer-binding site within the HIV-1 genomic RNA, thereby facilitating efficient annealing of tRNA^Lys,3^ to viral RNA prior to reverse transcription. Moreover, the host cell tRNA^Lys^ interacts with human KRS, Gag polyprotein, and GagPol precursor to form a packaging complex during HIV-1 assembly. **b** Viral infection specifically induces phosphorylation of EPRS at Ser990, which subsequently leads to the dissociation of EPRS from the MSC. The dissociated EPRS interacts with PCBP2 and blocks PCBP2-mediated MAVS ubiquitination, which in turn inhibits viral replication.

Interestingly, ARSs have become important players in a variety of viral infections (Fig. 3). Acquired immunodeficiency syndrome (AIDS) is an acquired defect of cellular immunity associated with infection by human immunodeficiency virus (HIV). Although this disease can be treated, it has no cure and has a major impact on health. Therefore, the prevention of HIV infection is very important^88^. During HIV-1 assembly, host cell tRNA^Lys,3^ as the primer for reverse transcription was selectively packaged into the virion by a specific interaction with human KRS, as well as viral Gag polyprotein and GagPol precursor^89–92^ (Fig. 3a). Strikingly, other ARSs were not detected in HIV-1, suggesting that KRS might be specifically incorporated into viral particles^89,93^. Recently, Duchon et al.^94^ observed that HIV-1 infection triggered the release of KRS from the MSC to form a free pool of KRS, which might be due to the specific phosphorylation of S207 in KRS (Fig. 3a). At the same time, the researchers found that the released KRS was partially transported to the nucleus. Interestingly, blocking this pathway by the addition of a MAPK/extracellular signal-regulated kinases (MEK) inhibitor in HIV-1-producing cells could reduce the infectivity of progeny virions, suggesting that HIV-1 utilized a dynamic MSC to enhance its own replication.

Actually, the incorporation of tRNA^Lys^ into the virion was closely related to its interaction with KRS^95^. When KRS in the infected cells was specifically inhibited, the resulting virus showed reduced tRNA^Lys^ packaging and tRNA^Lys,3^ annealing to viral RNA. In particular, the tRNA^Lys^ incorporation was dependent on the ability of KRS to bind to tRNA^Lys^, rather than its ability to aminoacylate tRNA^Lys^^96^. Furthermore, KRS bound to a tRNA-like element located near the primer-binding site within the HIV-1 genomic RNA, thereby facilitating efficient annealing of tRNA^Lys,3^ to viral RNA prior to reverse transcription^97–99^ (Fig. 3a). All together, these studies strongly elucidate that KRS plays a major role in HIV-1 assembly.

Additionally, Clarke et al.^100^ compared the cellular gene expression after brain infection with Japanese encephalitis virus (JEV) and West Nile virus (WNV) (two viruses that caused central nervous system disease) and reovirus (an unrelated neurotropic virus) by microarray analysis. In brains infected with all three viruses, many genes were upregulated, such as genes related to inflammation, IFN signaling, and the immune system, while genes related to glutamate signaling were downregulated. Notably, 14 ARSs were upregulated in JEV or WNV-infected brains, while none of these ARSs were upregulated after reovirus infection, suggesting that ARSs might be involved in the development of JEV or WNV-induced central nervous system disease. A 32-nucleotide RNA motif at the 3′ end of transmissible gastroenteritis coronavirus (TGEV) genome was found to interact with host EPRS and arginyl-tRNA synthetase (RRS)^101^. Since this RNA motif had high homology to the gamma interferon-activated inhibitor of translation (GAIT) element, it could bind to the GAIT complex and inhibit the translation of a chimeric mRNA comprising the RNA motif. Cells infected with TGEV harboring mutations in the 32-nucleotide RNA motif exhibited a more potent innate immune response mediated by the melanoma differentiation-associated gene 5 (MDA5) pathway, indicating that this RNA motif possibly inhibited the host immune response during TGEV infection. Recently, Lee et al.^102^ demonstrated that viral infection specifically induced phosphorylation of EPRS at Ser990, which subsequently led to the dissociation of EPRS from the MSC (Fig. 3b). The dissociated EPRS interacted with poly(rC)-binding protein 2 (PCBP2) and blocked PCBP2-mediated mitochondrial antiviral signaling protein (MAVS) ubiquitination, which in turn inhibited viral replication. Consistently, EPRS-haploid (*Eprs*^+/−^) mice exhibited extensive immunodeficiency, such as more severe viremia and delayed viral clearance.

#### ARSs in bacterial infection

Proteomic studies revealed that 26 proteins were significantly differentially expressed between the acute and convalescent phases of *Vibrio cholerae* O1 infection^103^. Through Gene Ontology (GO) analysis, the researchers demonstrated that these differentially expressed proteins were mainly related to innate immune responses, cytokine expression, and apoptosis. Interestingly, the levels of S100A8 and WRS were higher in the lamina propria cells during the acute stage of cholera, suggesting that these two proteins might play an important role in the intestinal inflammatory response in the early-stage cholera. Recently, by analyzing integrated transcriptome and metabolome datasets, Duffy et al.^104^ found that tuberculosis progression was associated with an immunometabolic profile, including tryptophan, cortisol, glutathione, and tRNA acylation networks. After infection by diverse pathogens, such as *Salmonella typhimurium*, *Staphylococcus aureus*, and respiratory syncytial virus, host monocytes rapidly secreted WRS^105^. The secreted WRS resulted in the production of cytokines in human and murine macrophages and increased levels of CD40, CD80, and CD86 on the cell surface, indicating that WRS could activate macrophages. Further studies found that WRS induced chemokine production and phagocytosis by binding to the TLR4-myeloid differentiation factor 2 (MD2) complex on macrophages.

Furthermore, The Shiga toxins produced by *Escherichia coli* induced KRS in macrophage-like differentiated THP-1 cells to dissociate from MSC and subsequently be secreted into extracellular space^106^. In turn, the secreted KRS could promote the production of pro-inflammatory cytokines in THP-1 cells, such as IL-8, IL-1β, and TNF-α. Differing from Shiga toxins, the acetyltransferase toxins produced by *Salmonella* Enteritidis and Typhimurium inhibited translation in macrophages by acetylation of aminoacyl-tRNAs, thereby inducing *Salmonella* persister formation during infection^107^. In conclusion, host ARSs not only participate in the HIV assembly, but also protect against bacterial and viral infections by modulating the immune responses, indicating that ARSs play an important role in infectious diseases.

### ARSs and tumor immunity

**Fig. 4:**
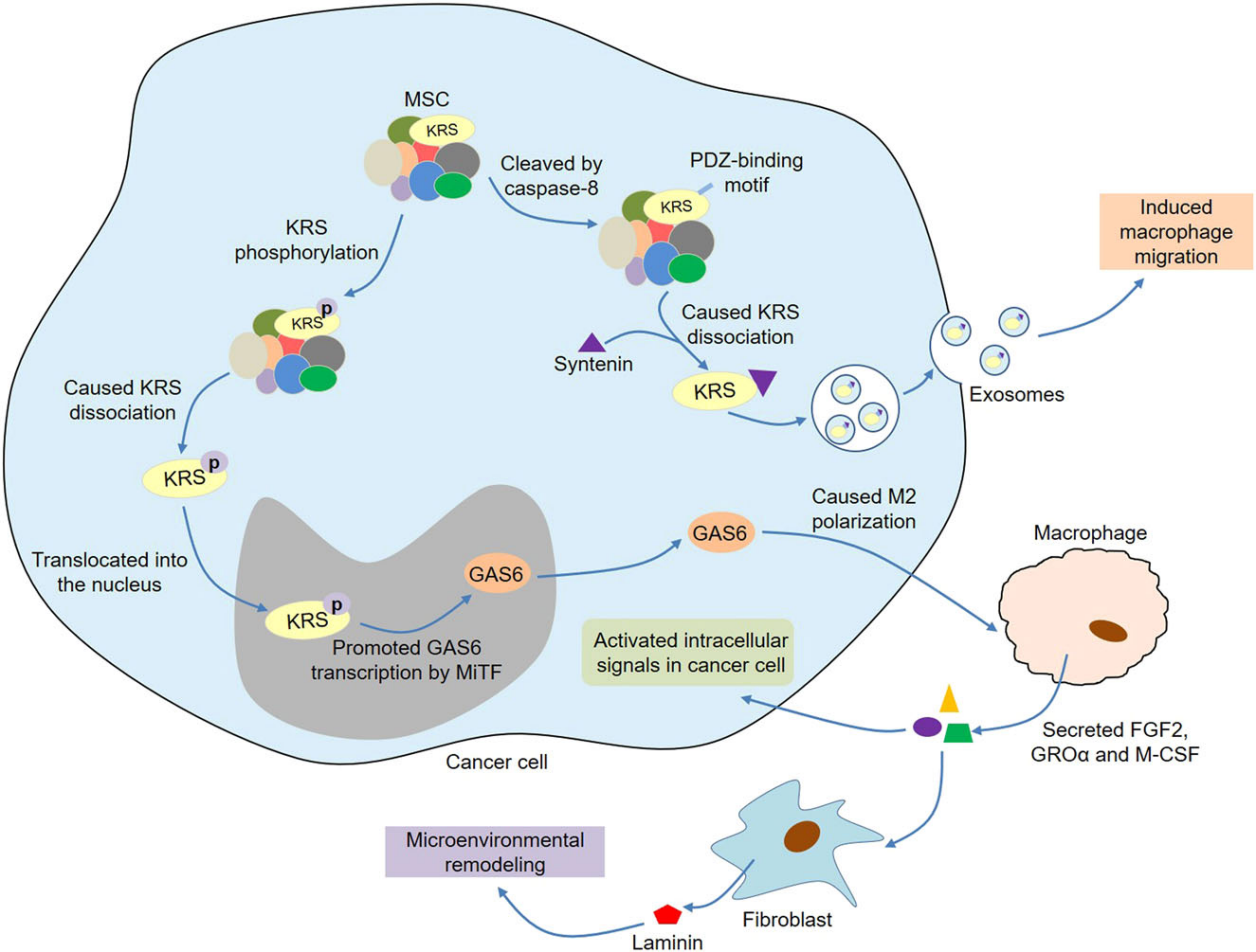
ARSs and tumor immunity. In tumor cells, a PDZ-binding motif at the C-terminus of KRS is exposed by cleaving the N-terminal by caspase-8. The exposed PDZ-binding motif binds to syntenin, which in turn promotes KRS dissociation from MSC and subsequent secretion into the extracellular space in the form of exosomes. The released exosomes induce macrophage migration and expression of various cytokines. Moreover, upon phosphorylation of the S207 residue of KRS in colon cancer cells, the KRS dissociates from MSC and translocates into the nucleus. Then, the nuclear KRS promotes GAS6 transcription by MiTF and thus causes M2 polarization of macrophages. M2 macrophages secrete FGF2, GROα, and M-CSF, which can not only activate intracellular signals in cancer cells but also promote laminin secretion by CAFs, leading to microenvironmental remodeling and cancer metastasis.

Strikingly, ARSs are closely related to tumor immunity (Fig. 4). Ovarian cancer cells could secrete ThrRS in response to cell stress, and ThrRS levels in patient cancer specimens were correlated with advancing disease stage and vascular endothelial growth factor (VEGF)^108^. It was of particular interest to find that ThrRS was over-expressed in infiltrating leukocytes, including neutrophils and plasma cells, within ovarian tumors. These data demonstrated that ThrRS might manipulate the tumor microenvironment through regulating angiogenesis and immune cell responses, thereby affecting tumor progression. Analogously, high expression of KRS might be present in gastric cancer cells and their infiltrating inflammatory cells, such as CD4^+^ T cells, macrophages/monocytes, and/or neutrophils^109^. Among them, patients with high KRS expression in cancer cells were associated with shorter overall survival of gastric cancer, while patients with high KRS expression in inflammatory cells were associated with longer overall survival. In addition, patients with high KRS expression in cancer cells accompanied by low or no KRS expression in inflammatory cells had significantly reduced survival rates. Conspicuously, Kim et al.^110^ systematically studied the secretion mechanism of KRS in colorectal carcinoma cells (Fig. 4). In tumor cells, a PDZ-binding motif at the C-terminus of KRS was exposed by cleaving the N-terminal by caspase-8. The exposed PDZ-binding motif bound to syntenin, which in turn promoted KRS dissociation from MSC and subsequent secretion into the extracellular space in the form of exosomes. The released exosomes could induce macrophage migration and expression of various cytokines. Interestingly, the exosomes containing very low KRS had stronger immunostimulatory activity than naked KRS, and HSP90, an immunostimulatory factor in exosomes, was positively correlated with KRS, indicating KRS might play a synergistic role with other inflammatory factors present in exosomes. Anthracyclines induced the translocation of calreticulin (CRT) to the surface of cancer cells, leading to immunogenic cell death^111^. Kepp et al.^112^ found that KRS also translocated to the surface of the cancer cells that stimulated by immunogenic death inducers and co-localized with CRT in lipid rafts. Moreover, KRS depletion inhibited CRT exposure, indicating that KRS was involved in the translocation of CRT in immunogenic cancer cell death.

A recent study by Adam et al.^113^ discovered that cancer cells could upregulate WRS in two different ways to adapt to the nutritional stress caused by tryptophan degradation. On the one hand, tryptophan depletion caused by high expression of indoleamine-2,3-dioxygenase-1 (IDO1) and tryptophan-2,3-dioxygenase (TDO2) in LN229 glioblastoma cells activated the general control non-derepressible-2 (GCN2) kinase, resulting in phosphorylation of eukaryotic translation initiation factor 2α (eIF2α) and the activation of activating transcription factor 4 (ATF4), which further upregulated WAS expression. On the other hand, tumor-infiltrating T cells jointly induced the expression of IDO1 and WRS in breast cancer, colon carcinoma, and B cell lymphoma by secreting IFN-γ. Upon phosphorylation of the S207 residue of KRS in colon cancer cells, the KRS molecule would dissociate from MSC and translocate into the nucleus^114^ (Fig. 4). Then, the nuclear KRS promoted growth arrest-specific 6 (GAS6) transcription by MiTF and thus caused M2 polarization of macrophages. M2 macrophages secreted multiple soluble factors, such as fibroblast growth factor 2 (FGF2), growth-regulated oncogene-α (GROα), and macrophage colony-stimulating factor, which not only activated intracellular signals in cancer cells but also promoted laminin secretion by cancer-associated fibroblasts, leading to microenvironmental remodeling and cancer metastasis. Furthermore, tumor cells could release Fas, an apoptotic ligand, which induced macrophages to secrete glycyl-tRNA synthetase (GRS)^115^. In turn, the secreted GRS bound to cadherin-6 in cancer cells, thereby enhancing phosphatase 2A (PP2A) activity. Finally, the activated PP2A inhibited ERK signaling via dephosphorylation of ERK, thereby suppressing tumorigenesis. Overall, ARSs are active participants in tumor immunity. On the one hand, tumor cells regulate the functions of immune cells by secreting ARSs. On the other hand, tumor-related immune cells can also secrete ARSs, thereby affecting tumor development.

## Conclusion and future perspective

All in all, ARSs have emerged as multifaceted molecules participating in immune regulation and immune diseases. First, pathogen ARSs can induce immune responses in the host, which in turn serve as targets for anti-infection. Secondly, ARSs are involved in the maturation, transcription, activation, and recruitment of immune cells, thus playing a crucial role in the development of immune cells. More importantly, ARSs act as regulators and signaling molecules in various immune diseases, such as autoimmune diseases, infectious diseases, and tumor immunity (Table 1). ARSs mainly contribute to the occurrence and development of autoimmune diseases as autoantigens. In addition, host ARSs not only participate in the HIV assembly, but also protect against bacterial and viral infections, indicating that ARSs are closely related to infectious diseases.

**Table 1:** Roles of ARSs in immune diseases.

Immune diseases	ARSs	Effects	Mechanisms	References
Autoimmune diseases	ARSs served as autoantigens in ASSD patients	Anti-ARS autoantibody specificity was related to the clinical features, disease severity, and even survival of ASSD patients	-	^65–71^
	HisRS and AsnRS	Acted as chemoattractants for leukocytes	The unique N-terminal extension domain of AsnRS was associated with the CCR3-mediated chemotactic activity	^72,73^
	HisRS	Provoked myositis in mice	By MyD88-dependent TLRs	^74^
	ARSs served as autoantigens in ASSD patients	NK cells might contribute to the development of ASSD	NK cells had abnormal phenotypic characterization and function	^75^
	HisRS in polymyositis patients	PBMC-derived APCs and DCs mediated peripheral blood T cell proliferation triggered by HisRS	–	^76^
	HisRS	Murine HisRS induced autoreactive B and T cells targeting its own epitopes	–	^77^
	HisRS in ASSD patients	Presence of HisRS-specific CD4^+^ T cells in the blood and lung of ASSD patients	–	^78^
	HisRS in ASSD patients	B cell homeostasis was impaired in ASSD patients with anti-Jo-1	The frequency of CD19^+^CD27^+^ memory B cells was decreased, while the frequency of CD19^+^CD27^−^ naive B cells was increased	^79^
	HisRS in ASSD patients	Might be responsible for the pathogenicity of anti-Jo-1 autoantibodies	Jo-1-positive patients showed an Fc-glycan profile with less bisected and afucosylated glycans, and the Fc-glycan profile features were correlated with certain clinical and diagnostic information	^80^
	ARSs were dysregulated in certain autoimmune diseases	Associated with the development of autoimmune diseases, such as multiple sclerosis, rheumatoid arthritis, and systemic lupus erythematosus	–	^81–85^
	WRS	Related to the pathogenesis of Graves’ disease	The high levels of WRS in CD4^+^ T cells abolished IDO-mediated immunosuppression from DCs	^87^
Infectious diseases	KRS	Involved in HIV-1 assembly	Interacted with tRNA^Lys^, Gag polyprotein, and GagPol precursor to form a packaging complex	^89–92^
	KRS	Associated with the infectivity of progeny virions	HIV-1 infection triggered the release of KRS from the MSC, and then the released KRS was partially transported to the nucleus	^94^
	KRS	Associated with the incorporation of tRNA^Lys^ into the virion	The tRNA^Lys^ incorporation was dependent on the ability of KRS to bind to tRNA^Lys^, rather than its ability to aminoacylate tRNA^Lys^	^95,96^
	KRS	Facilitated efficient annealing of tRNA^Lys,3^ to viral RNA prior to reverse transcription	KRS bound to a tRNA-like element located near the primer-binding site within the HIV-1 genomic RNA	^97–99^
	14 ARSs were upregulated in JEV or WNV-infected brains	Involved in the development of JEV or WNV-induced central nervous system disease	–	^100^
	EPRS and RRS	Related to the host immune response during TGEV infection	A 32-nucleotide RNA motif at the 3′ end of TGEV genome interacted with EPRS and RRS. Moreover, it bound to the GAIT complex and inhibited the translation of a chimeric mRNA comprising the RNA motif	^101^
	EPRS	Inhibited viral replication	Interacted with PCBP2 and then blocked PCBP2-mediated MAVS ubiquitination	^102^
	WRS levels were higher in the lamina propria cells during the acute stage of cholera	Possibly involved in the intestinal inflammatory response in the early-stage cholera	–	^103^
	WRS	Activated macrophages during infection	Bound to the TLR4-MD2 complex on macrophages	^105^
	KRS	Involved in the immune responses induced by the Shiga toxins produced by *Escherichia coli*	The Shiga toxins induced KRS secretion from macrophage-like differentiated THP-1. In turn, the secreted KRS promoted the production of pro-inflammatory cytokines in THP-1 cells	^106^
Tumor immunity	ThrRS	Manipulated the tumor microenvironment through regulating angiogenesis and immune cell responses	ThrRS levels were correlated with VEGF, and it was over-expressed in infiltrating leukocytes	^108^
	KRS	Might be an independent prognostic marker for gastric cancer patients	High expression of KRS might be present in gastric cancer cells and their infiltrating inflammatory cells	^109^
	KRS	Associated with inflammation in cancer	Caspase-8 mediated the release of KRS from tumor cells, and the released KRS induced macrophage migration and expression of various cytokines	^110^
	KRS	Associated with the immunogenic cancer cell death	Involved in the translocation of CRT to the surface of cancer cells	^111,112^
	WAS	Adapted human cancer cells to tryptophan degradation	GCN2-eIF2α-ATF4 signaling and IFN-γ jointly mediated the upregulation of WRS in response to tryptophan degradation in tumor cells	^113^
	KRS	Led to microenvironmental remodeling and cancer metastasis	KRS-expressing colon spheroids induced M2 macrophage polarization and then the secretion of multiple soluble factors, which activated intracellular signals in cancer cells and promoted laminin secretion by CAFs	^114^
	GRS	Suppressed tumorigenesis	Tumor cells induced macrophages to secrete GRS by releasing Fas. In turn, the secreted GRS inhibited ERK signaling in cancer cells	^115^

*ARSs* aminoacyl-tRNA synthetases, *ASSD* antisynthetase syndrome, *HisRS* histidyl-tRNA synthetase, *AsnRS* asparaginyl-tRNA synthetase, *CCR3* CC chemokine receptor 3, *MyD88* multiple myeloid differentiation primary response gene 88, *TLR* Toll-like receptor, *NK cells* natural killer cells, *PBMC* peripheral blood mononuclear cell, *APCs* antigen-presenting cells, *DCs* dendritic cells, *WRS* tryptophanyl-tRNA synthetase, *IDO* indoleamine-2,3-dioxygenase, *KRS* lysyl-tRNA synthetase, *HIV-1* human immunodeficiency virus-1, *MSC* multisynthetase complex, *JEV* Japanese encephalitis virus, *WNV* West Nile virus, *EPRS* glutamyl-prolyl-tRNA synthetase, *RRS* arginyl-tRNA synthetase, *TGEV* transmissible gastroenteritis coronavirus, *GAIT* gamma interferon-activated inhibitor of translation, *PCBP2* poly(rC)-binding protein 2, *MAVS* mitochondrial antiviral signaling protein, *MD2* myeloid differentiation factor 2, *ThrRS* threonyl-tRNA synthetase, *VEGF* vascular endothelial growth factor, *CRT* calreticulin, *GCN2* general control non-derepressible-2, *eIF2α* eukaryotic translation initiation factor 2α, *ATF4* activating transcription factor 4, *IFN-γ* interferon-γ, *CAFs* cancer-associated fibroblasts, *GRS* glycyl-tRNA synthetase, *ERK* extracellular signal-regulated kinase.

In fact, the search for new therapeutic agents from the perspective of ARSs biology has received great attention^3,116,117^. These candidate agents mainly act on three aspects related to ARSs, including the catalytic sites of ARSs, the extracellular activities of secreted ARSs, and the proteins that interact with ARSs. So far, the development of drug candidates for cancer, inflammation, fibrosis, and neural disorders has progressed relatively smoothly. Of note, febrifugine and its derivatives inhibited the activity of prolyl-tRNA synthetase (ProRS) by binding to EPRS, thereby promoting inflammation regression^118,119^. Cyclic peptides could bind to a site proximal to the helix 4 of capsid C-terminal domain, thereby inhibiting the interaction of human KRS with capsid, suggesting that the cyclic peptides might be potential drugs against HIV-1 infection^120^. Moreover, several animal models reproduced the characteristics of human ASSD, which might serve as tools for preclinical research of this autoimmune disease^121–123^.

However, some issues remain to be resolved. In-depth research is needed on the safety and drug resistance of potential anti-infective compounds that target pathogen ARSs. Meanwhile, the exploration of molecular mechanisms is warranted to clearly illuminate the relationships between ARSs and immune processes. Nevertheless, the understanding of ARSs in the immune context provides new ideas for the treatment of immune diseases.
